# Complex Traumatic Jejunal Perforation With Mesenteric Tear and Intussusception: A Case Report

**DOI:** 10.7759/cureus.61852

**Published:** 2024-06-06

**Authors:** Khushbu Vaidya, Pankaj Gharde, Raju K Shinde, Mihir Patil, Somya Goel

**Affiliations:** 1 General Surgery, Jawaharlal Nehru Medical College, Datta Meghe Institute of Higher Education and Research, Wardha, IND

**Keywords:** multidisciplinary management, exploratory laparotomy, mesenteric tear, jejunal perforation, hollow viscus perforation, blunt abdominal trauma

## Abstract

Blunt abdominal trauma can result in a spectrum of injuries, ranging from superficial contusions to severe hollow viscus perforations. We present the case of a 52-year-old male involved in a bicycle-truck collision, leading to complex intra-abdominal injuries. The patient presented with acute abdominal pain and signs of peritonitis, prompting urgent diagnostic workup and surgical intervention. Imaging studies revealed pneumoperitoneum, free fluid, and multiple rib fractures indicative of significant trauma. Exploratory laparotomy unveiled a perforated jejunal loop with an associated mesenteric tear and intussusception, necessitating segmental bowel resection and repair. Histopathological analysis confirmed acute hemorrhagic inflammation consistent with traumatic perforation. This case highlights the challenges and complexities associated with blunt abdominal trauma, emphasizing the importance of prompt recognition, multidisciplinary management, and surgical intervention in optimizing patient outcomes.

## Introduction

Blunt abdominal trauma is a critical condition that often poses significant diagnostic and management challenges and is frequently encountered in patients involved in traffic accidents, falls, or assaults. The abdomen, being less protected than other body parts, is particularly susceptible to injury from blunt force. Such trauma can lead to various injuries, including solid organ injury (liver, spleen, kidneys), hollow viscus injury (small and large bowel, bladder), and vascular injury [1]. Hollow viscus injuries (HVI) are among the most challenging consequences of blunt abdominal trauma to diagnose, primarily due to their non-specific clinical and imaging findings. They are identified in approximately 1-5% of blunt abdominal traumas [2]. The Centers for Disease Control and Prevention (CDC) states that HIV tests have 99.6% specificity, which means that for every 1,000 people without HIV who take the test, 996 will receive true negative results, and four may receive a false positive [2-3]. The most commonly injured parts of the gastrointestinal tract in blunt trauma are the small intestine, colon, and duodenum [3]. The diagnostic difficulty arises because initial symptoms and signs may be subtle or absent, and delays in diagnosis can lead to significant morbidity and mortality due to sepsis and peritonitis [4].

Perforation of the small intestine, as seen in our case, is particularly concerning as it is associated with high infection rates and requires prompt surgical intervention. Clinical assessment remains crucial, including careful monitoring of vital signs and physical examination findings [5]. Moreover, mesenteric tears, which may accompany small bowel perforations, add another layer of complexity. They can lead to compromised blood supply and result in bowel ischemia or infarction if not promptly addressed [6]. Intussusception in adults, although rare, can occur as a consequence of trauma and may present with similar radiologic features to other types of bowel injury [7].

## Case presentation

A 52-year-old male was admitted to the emergency department following a collision between his bicycle and a truck, which resulted in direct impact trauma from the bicycle handle to his chest and abdomen. Upon presentation, the patient complained of severe, diffuse abdominal pain without any prior history of significant medical issues, head injuries, or episodes of unconsciousness. His initial vital signs just after the admission to the emergency department indicated a tachycardic state with a pulse of 130 bpm, hypotension at 104/60 mmHg, and oxygen saturation at 96% on 3 L/min supplemental oxygen. Physical examination revealed abdominal distension, tenderness, and guarding suggestive of an acute abdominal crisis.

**Figure 1:**
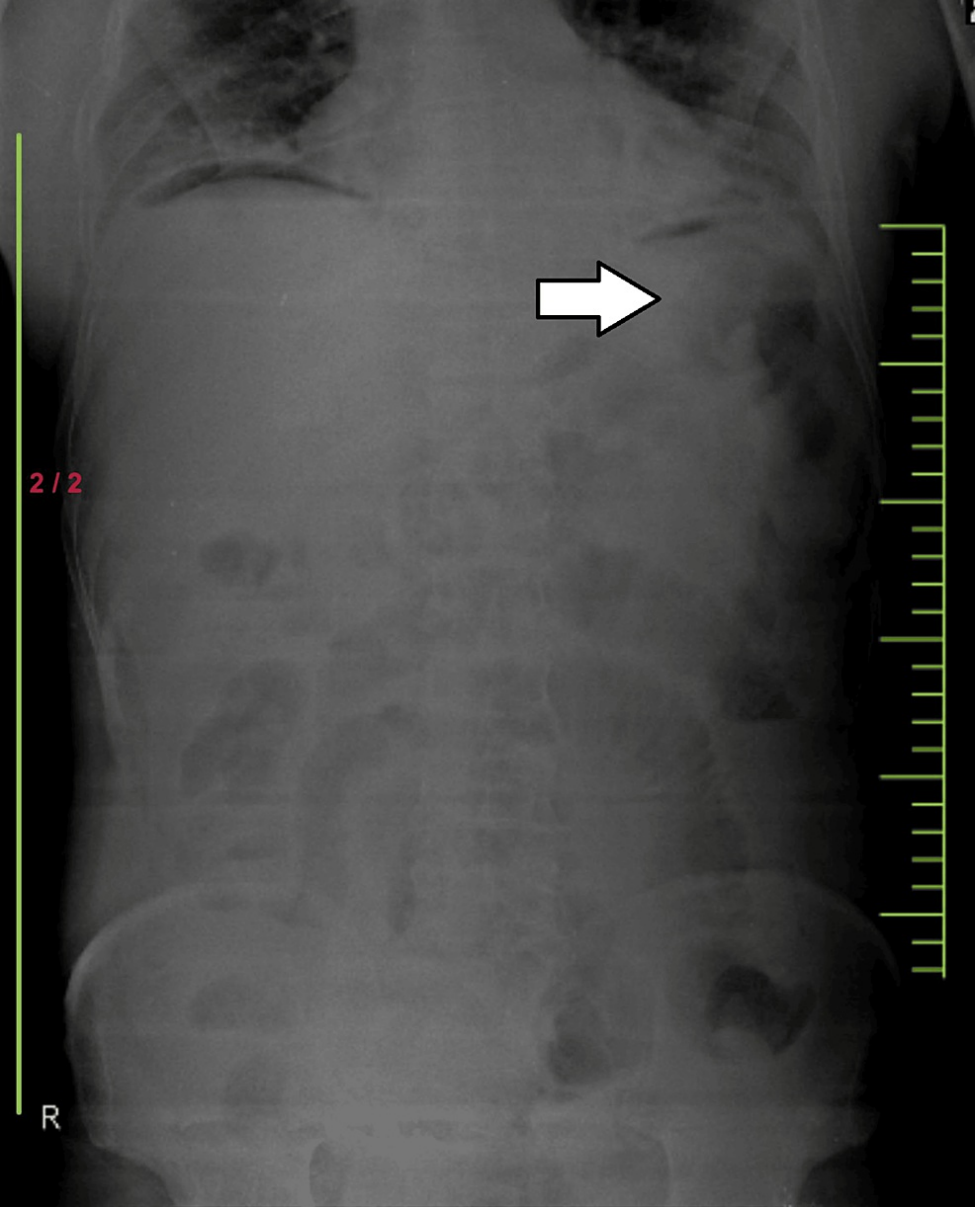
Signs consistent with pneumoperitoneum

Initial diagnostic investigations included an abdominal X-ray, which showed signs consistent with pneumoperitoneum, indicating a possible hollow viscus perforation as shown in Figure 1. An abdomen ultrasound identified free fluid consistent with ascites in perihepatic and perisplenic regions. A CT scan of the abdomen and pelvis was performed to further evaluate the extent of injuries, revealing small pockets of air in the preduodenal and periportal regions and moderate free fluid. These findings highly suggest a hollow viscus injury involving the small bowel. A chest CT scan was also conducted due to the nature of the trauma, which revealed pneumothorax, subcutaneous emphysema, and multiple rib fractures from the right fifth to eighth ribs.

**Figure 2:**
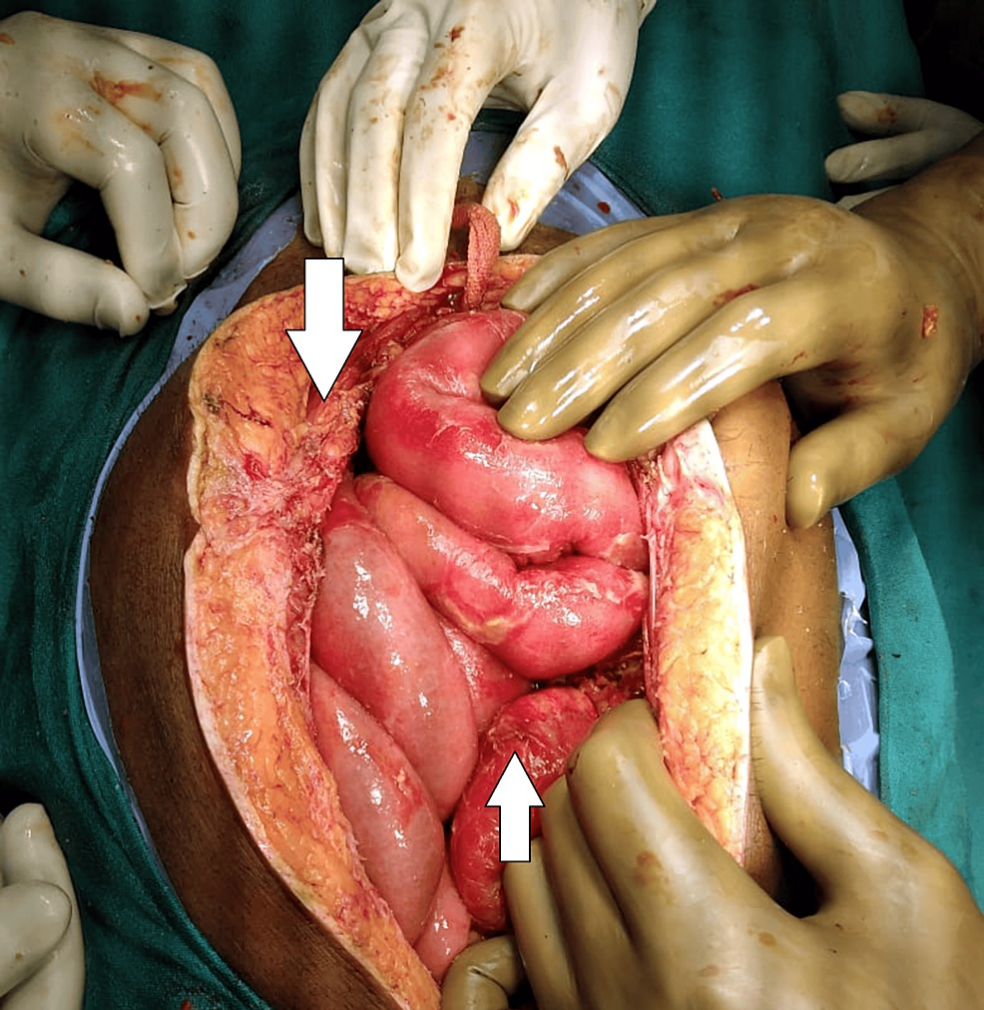
Perforated jejunal loop with a mesenteric tear

Given the clinical and radiological evidence of intra-abdominal injury, the patient was urgently taken to the operating room for an exploratory laparotomy. During surgery, a perforated jejunal loop with a mesenteric tear was discovered as shown in Figure 2.

**Figure 3:**
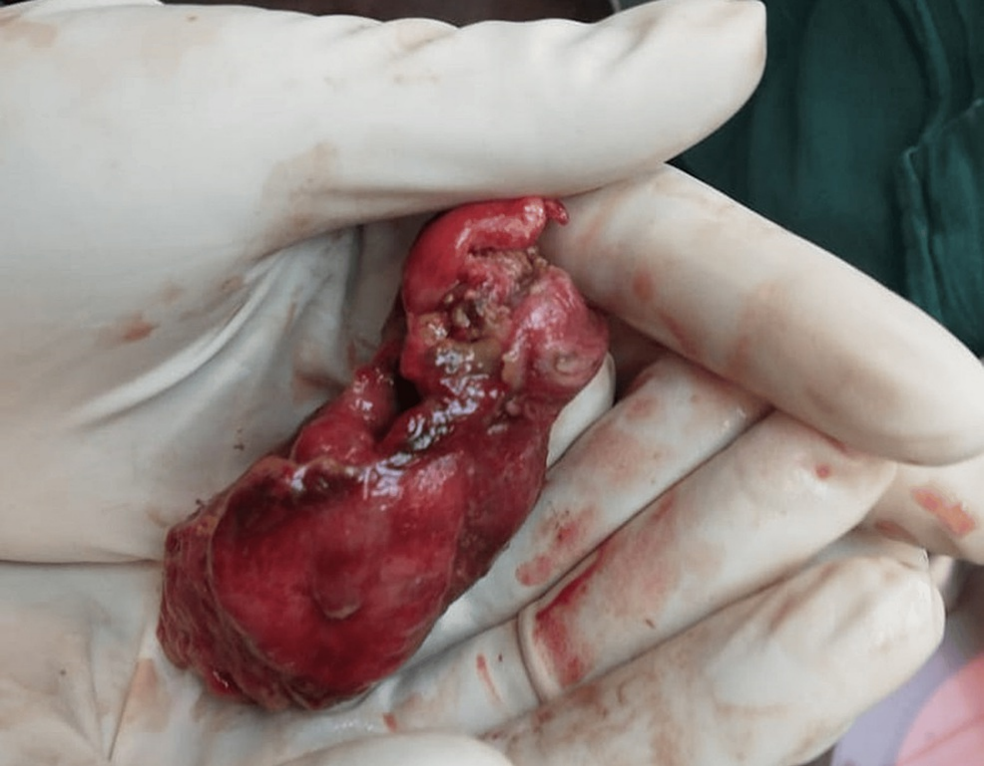
Evacuated hematoma

Additionally, a segment of the small bowel was found intussuscepting through the perforation site, complicating the injury pattern. Segmental jejunal resection was performed with primary anastomosis. The colon serosa that had been damaged was repaired via suturing, and a necrotic section of the colon necessitated partial colectomy. Adjacent to the primary lesion in the small bowel mesentery, a benign hematoma was evacuated as shown in Figure 3.

Histopathological examination of the resected bowel segments confirmed acute hemorrhagic inflammation consistent with traumatic perforation. The complex nature of the patient’s injuries required multidisciplinary management, including surgical intervention, intensive postoperative care, and nutritional support to address the immediate and secondary complications of such severe trauma. This case highlights the critical nature of rapid assessment and intervention in patients presenting with blunt abdominal trauma, the complexity of injuries that can occur, and the importance of a thorough and multidisciplinary approach to management to optimize patient outcomes.

## Discussion

The presented case exemplifies the complexity and severity of intra-abdominal injuries resulting from blunt trauma, necessitating prompt recognition and multidisciplinary management. Blunt abdominal trauma encompasses a broad spectrum of injuries, ranging from mild contusions to life-threatening hollow viscus perforations, as seen in this patient [8]. The occurrence of both bowel perforation and mesenteric tear, complicated further by bowel intussusception, represents a rare and challenging scenario in trauma care [9]. A lead point is found in around 80-90% of cases, even though the annual incidence of adult intussusception is very low [9]. While solid organ injuries often dominate discussions on blunt abdominal trauma, hollow viscus injuries, particularly involving the small bowel, present unique diagnostic and therapeutic challenges due to their nonspecific clinical manifestations and subtle radiological findings [9].

Diagnostic modalities such as abdominal X-ray, ultrasound, and computed tomography are crucial in assessing patients with suspected hollow viscus injuries. In this case, the presence of pneumoperitoneum on X-ray and free fluid on ultrasound and CT scan aided in identifying the extent and severity of intra-abdominal injuries, prompting the need for urgent surgical intervention. [10]. Surgical exploration remains the gold standard for definitive diagnosis and management of hollow viscus injuries. The intraoperative findings of a perforated jejunal loop with associated mesenteric tear and bowel intussusception underscore the complexity of the patient's injuries, necessitating segmental bowel resection, serosal repair, and partial colectomy [11].

Histopathological examination of the resected bowel segments confirmed acute hemorrhagic inflammation consistent with traumatic perforation, highlighting the importance of corroborating clinical and radiological findings with histological evidence to guide patient management and prognosis [12]. This case emphasizes the critical role of prompt recognition and multidisciplinary management in optimizing outcomes for patients with blunt abdominal trauma. A comprehensive approach involving trauma surgeons, radiologists, anesthesiologists, and intensive care specialists is essential to address the varied and often life-threatening consequences of intra-abdominal injuries [13].

## Conclusions

In conclusion, the presented case underscores the intricate nature of intra-abdominal injuries secondary to blunt trauma, exemplified by the rare occurrence of perforated bowel with mesenteric tear and intussusception. Through prompt recognition, accurate diagnosis, and multidisciplinary management, favorable outcomes were achieved for the patient despite the complexity of his injuries. This case highlights the critical role of surgical intervention guided by advanced imaging modalities and histopathological confirmation in optimizing patient outcomes. Moving forward, continued emphasis on comprehensive trauma care protocols and interdisciplinary collaboration is paramount to effectively address the varied and often life-threatening consequences of blunt abdominal trauma, ensuring timely and appropriate management for patients presenting with similar challenging injuries.
